# Anti‐inflammatory ethosomal nanoformulation in combination with iontophoresis in chronic wound healing: An ex vivo study

**DOI:** 10.1049/nbt2.12069

**Published:** 2021-10-18

**Authors:** Reza Mombeiny, Shima Tavakol, Mostafa Kazemi, Mehdi Mehdizadeh, Akbar Hasanzadeh, Mohammad Karimi Babaahmadi, Ali Abedi, Peyman Keyhanvar

**Affiliations:** ^1^ Nanotechnology Research Center Ahvaz Jundishapur University of Medical Sciences Ahvaz Iran; ^2^ Cellular and Molecular Research Center Iran University of Medical Sciences Tehran Iran; ^3^ Cellular and Molecular Research Center Faculty of Advanced Technologies in Medicine Department of Anatomy Iran University of Medical Sciences Tehran Iran; ^4^ Department of Medical Biotechnology Faculty of Advanced Medical Sciences Tabriz University of Medical Sciences Tabriz Iran; ^5^ Department of Life Sciences Engineering Faculty of New Sciences and Technology University of Tehran Tehran Iran; ^6^ Stem Cell Research Center Stem Cells and Regenerative Medicine Institute Tabriz University of Medical Sciences Tabriz Iran; ^7^ Stem Cell and Regenerative Medicine Institute (SCARM) Tabriz University of Medical Sciences Tabriz Iran; ^8^ Technology and Society Network (CKTSN) Universal Scientific Education and Research Network (USERN) Tehran Iran

**Keywords:** chronic wound healing, ethosome, hydrocortisone 17‐butyrate, nano‐carrier, nanoparticle, transdermal delivery

## Abstract

Prescription of anti‐inflammatory drugs may be considered as a promising strategy in chronic wound healing where the inflammatory disturbance has delayed the healing process. It seems that hydrocortisone 17‐butyrate (HB17) would be promising in the form of a nano‐formulation to enhance drug delivery efficacy. In the present study, transdermal delivery of nano‐HB17 in combination with iontophoresis was investigated ex vivo. Ethosomal‐HB17 was synthesised using lecithin, ethanol and cholesterol with a different ratio by hot method. The negative ethosomal‐HB17 particle size was around 244 ± 4.3 nm with high stability of up to 30 days. Additionally, evaluated entrapment efficiency of HB17 in ethosomes by high performance liquid chromatography was 40.6 ± 2.21%. Moreover, the permeation speed and amount of H17B in complete‐thickness rat skin in the presence and absence of iontophoresis showed that the penetration of free H17B and ethosomal‐H17B formulations were zero and 7.98 μg/cm^2^ in 120 min, respectively. Whereas in the case of applying iontophoresis, permeation amount obtained was zero and 19.69 μg/cm^2^ in 30 min in free H17B and ethosomal‐H17B formulations, respectively. It has been concluded that transdermal delivery of ethosomal‐H17B is an effective strategy to enhance drug delivery and it will be improved when it is combined with iontophoresis.

## 1 INTRODUCTION

Wound healing is a complicated [1] process in which the inflammatory phase plays a critical role in the progression and decline of wound healing in acute and chronic wounds, respectively [2]. The commercial market related to wound care products and scarring are US$15 and US$12 billion, respectively [3].

A chronic wound is a wound that has not healed during a predictable time same as other wounds. In brief, at the first stage, inflammatory mechanisms are triggered to clear the pathogens and then, after about three days, the proliferation phase of wound healing by fibroblasts through the secretion of collagen and other substances has started to make tissue scaffold. Following angiogenesis, endothelial cells transit to the remodelling (maturation) phase during 2–3 weeks [3]. Several factors resulted in delayed wound healing such as oxygen tension (20 mmHg instead of 5 mmHg), protein [4] and vitamin A, C and Zinc uptake [5], temperature and moisture, etc. A chronic wound is characterized by the depletion of EGF, FGF, KGF, VEGF, PDGF, TGF‐β growth factors and elevated IL1 and 6 and TNF‐α [6]. Therefore, the substitution of just one factor is not enough to restore complete wound healing. There are several methods to address chronic wound healing, including ultrasound, electromagnetic therapy, vacuum‐assisted closure therapy and skin graft [7, 8, 9, 10].

It is demonstrated that anti‐inflammatory medications through the down‐regulation of precollagen mRNA [11], TGF‐β and IGF‐1 [12] delay wound healing; however, in chronic wounds in which inflammatory mechanisms are disturbed, anti‐inflammatory drugs such as corticosteroids promote wound healing [2]. In this regard, it seems that the applying of anti‐inflammatory medication in chronic wounds would be of vital importance.

There are several dosage forms for drug delivery systems. Some alternative routes such as intravenous infusion, buccal and transdermal routes can improve drug bioavailability. These routes not only eliminate first‐pass by hepatic, but also retain constant, prolonged and therapeutically effective drug levels in the body while these alternative approaches are invasive and painful [13]. In this way, transdermal delivery has been developed as one of the attractive alternative approaches as compared to oral and injection drug deliveries.

For a long time, the penetration of pharmaceutical substances through the skin surface has been known as a different form of systemic medication [13, 14, 15, 16]. The penetration of a drug through the skin may exhibit drug sustained release in a controlled manner and leads to a decrease in the drug's dosage [14]. In contrast, one of the most important challenges associated with this drug delivery route is low permeability of medicine, preventing the progression of this strategy. Regarding this, many efforts were being made to remove this challenge, including the physical and chemical modification methods. For instance, many devices were used to increase the penetration of the drug on the surface of skin as physical modification methods [17, 18, 19, 20, 21]. Iontophoresis was commonly applied as an electrical device with a continuous low voltage current to enhance transdermal delivery [22, 23, 24].

Investigations suggest two pathways to transport the drug from the stratum corneum barrier: i) Intracellular and ii) trans accessory [25, 26]. In this regard, studies showed that the transcellular pathway was a possible way for the permeation of the therapeutic agents through skin, using a space about 50 to 70 nm in diameter [27]. Accordingly, drug‐loaded nanocarriers are eligible for passing through this space and several nanocarriers have been investigated to achieve this goal. Recently, ethosomes were introduced as a novel carrier with nano to micron sizes, composed of phospholipids and ethanol/isopropyl alcohol (relatively with high concentration) in water. These carriers are flexible lipid vesicles, which lead to transferring the drug to deep layers of the skin and/or the systemic circulation [28]. It seems that the combination of phospholipids and high concentration of ethanol plays a principal role in the deeper penetration of a medicine agent in the skin [29]. Since ethosome is known as a proper carrier for both hydrophobic and hydrophilic drugs, it was preferred for transferring a variety of drugs through the surface of the skin as topical forms [30]. Accordingly, a novel form of drug delivery system based on ethosome has been reported to cross the ketoconazole through the skin [31]. Moreover, reports indicate that nanoethosomal carriers enhance the delivery of clonazepam as a transdermal system [32]. In this regard, studies have shown that the combination of nano‐carrier and iontophoresis technique has a synergistic effect on the delivery of a very low penetrating therapeutic agent [25, 33].

In this study, hydrocortisone 17‐butyrate (H17B) as an anti‐inflammatory drug‐loaded ethosome in a nano size range was prepared and characterized by several techniques. In addition, entrapment efficiency, as a significant factor for optimizing the formulation of ethosomes, was calculated using HPLC system. Subsequently, transdermal delivery of H17B by the combination of ethosomes and iontophoresis into the rat skin was evaluated by the HPLC system.

## 2 EXPERIMENTAL SECTION

### 2.1 Material

Hydrocortisone 17‐butyrate, phospholipid, cholesterol, and absolute ethanol were purchased from Sigma. Distilled water was also used throughout the study.

### 2.2 Preparation of hydrocortisone 17‐butyrate‐loaded ethosome

Ethosome colloidal suspensions were prepared by the hot method as previously reported [40]. Typically, a specific amount of phospholipid was dissolved in 5 ml of absolute ethanol (99.9%), which presented a colloidal solution that was completely dissolved by using sonication. Subsequently, cholesterol (5% W relative to phospholipid) was added to the solution and sonicated into the ultrasonic bath for 5 min (solution 1). Then, 5 mg of H17B was dissolved in 5 ml absolute ethanol in a separate test tube and sonicated into the ultrasonic bath for 5 min until a homogeneous solution was obtained (solution 1). In other words, the following agents were constant in all formulations; 10 ml of ethanol (40%), 25 ml water and a constant ratio between concentrations of lecithin/cholesterol (cholesterol 5% of lecithin). However, in all formulations, the lecithin concentration was variable. After this, the solution 1 was added to the solution 2, and the final solution (as organic phase) was dispersed in an aqueous phase dropwise, while using a homogeniser at 45°C. Table 1 shows the specific amount of the substance such as alcohol, phospholipid, cholesterol and water in this work.

**TABLE 1:** Ethosomal formulation characteristics

Ethosomal formulation	Lecithin (mg)	Particle size (nm)	pH	Turbidity (NTU)	Stability (days)
Etho1	2.5	462 ± 8.7	7.5 ± 0.26	9.3 ± 1.04	15
Etho2	5	244 ± 4.3	7.3 ± 0.17	14.0 ± 1.50	45
Etho3	10	281 ± 8.1	4.9 ± 0.36	21.5 ± 1.50	25
Etho4	20	132 ± 6.2	4.0 ± 0.26	50.0 ± 8.88	20

### 2.3 Nanoparticle characterisation

#### 2.3.1 Particle size and morphological studies

**FIGURE 1:**
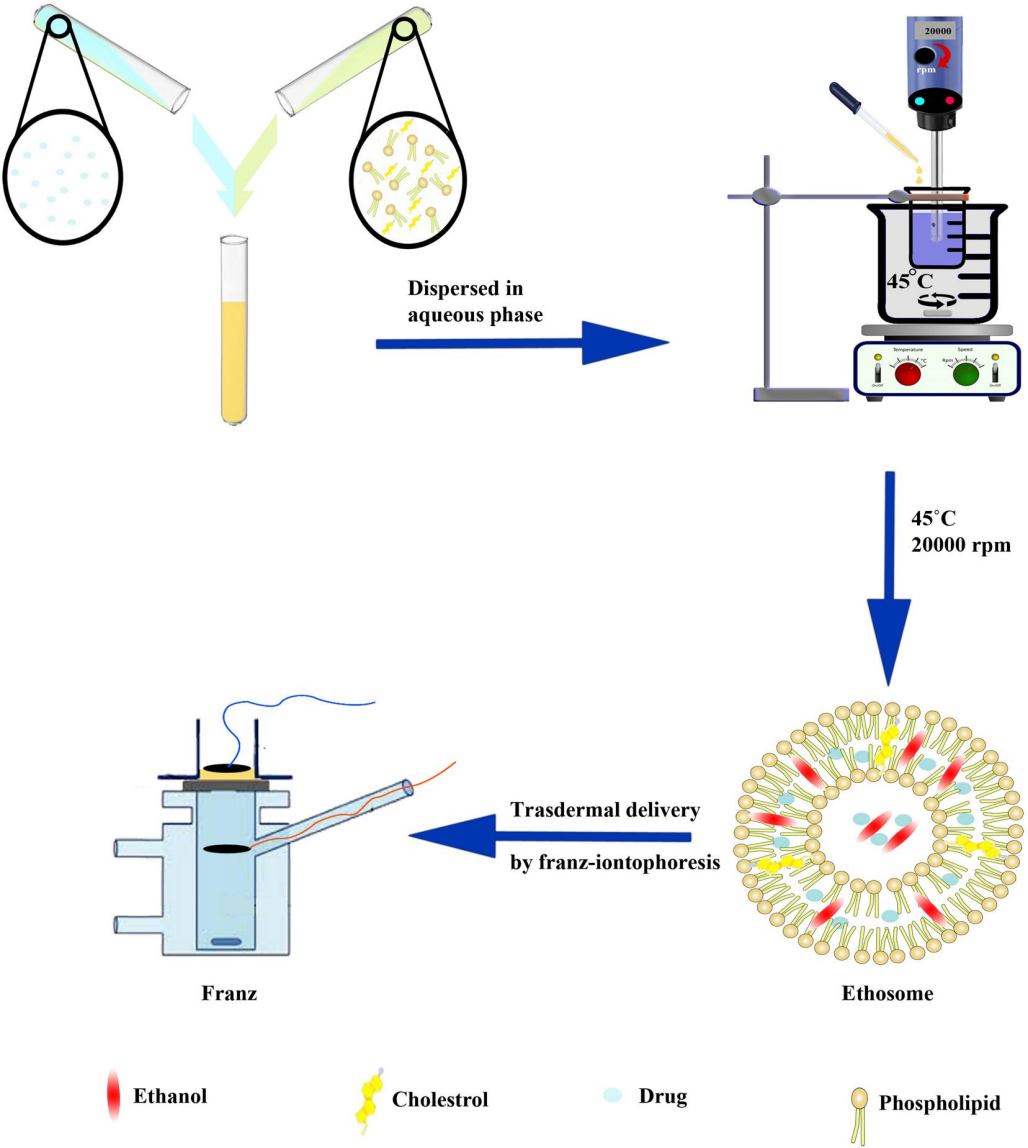
Ethosomal preparation and transdermal delivery by Franz‐iontophoresis

Surface morphology of ethosome was visualised using scanning electron microscopy (SEM, KYKY‐EM3200). To prepare the sample, a drop of colloidal ethosomes was placed onto a glass slide, and then coated by gold for visualising. For TEM imaging (TEM, CM30 3000Kv), some specimens of synthesised nano‐carrier were prepared by ultrasonic dispersion of the nano‐carrier in ethanol, and the suspensions were dropped onto a glass slide. The assay was performed in triplicate and the values provided are the normalized mean ± SD of three independent experiments (Figure 1).

#### 2.3.2 Size distribution and stability evaluations of ethosome

The size distribution of ethosomes was characterised by the DLS technique using a computerised inspection system (MALVERN Zen3600) with DTS® (nano) software. To determine the stability of synthesised nano ethosomes, the formulation was stored in a standard condition for up to 30 days and particle size measurement carried out with DLS analysis. The assay was performed in triplicate, and the values provided are the normalized mean ± SD of three independent experiments.

#### 2.3.3 Zeta potential

Zeta potential was performed by Zeta sizer (MALVERN Zen3600) to investigate the surface charge of ethosomes. The acidity of all the samples was first neutralised, and they were diluted by 10 mM NaCl. Zeta potential was evaluated at 25°C with a −50 mV latex standard. The assay was performed in triplicate and the values provided are the normalized mean ± SD of three independent experiments.

#### 2.3.4 Entrapment efficiency (EE)

The best method to evaluate the entrapment and loading efficacy of nanocarriers is ultrafiltration and in this study, we used it [34, 35]. The EE of nanoethosomal H17B was assessed using the HPLC system, (YL9100). The mobile phase was methanol–water‐ acetonitrile (130:80:1v/v). The analytical method was accredited for linearity, accuracy and precision. The standard curve of hydrocortisone 17‐butyrate was drawn. In this method, H17B loaded‐ethosome was centrifuged in an amicon tube 10 KD for 5 min at 1500 rpm to separate the unentrapped drug and the supernatant was analysed by the HPLC system. Additionally, the remaining ethosomes were lysed using Triton X 100 (0.1% v/v) and was analysed. EE was declared as a percentage of total H17B entrapped. Finally, the entrapment efficiency was calculated by applying the following equation:

(1) $$\frac{C-T}{C}\times 100\;$$

where *C* is the total amount of drug and *T* is the free drug amount, which was detected only in the supernatant media. The assay was performed in triplicate and the values provided are the normalized means ± SD of three independent experiments.

### 2.4 Ex‐vivo study

#### 2.4.1 Permeation analysis of H17B‐ethosomes into the skin with and without iontophoresis

Ex‐vivo analysis was performed on rat skin using a Franz diffusion cell. The receptor cell volume and effective penetration region were 6.5 ml and 3 cm^2^, respectively. Briefly, following shaving and cutting of rats’ skins, the rat skins were washed and assessed for accuracy. Subsequently, ethosome solutions were poured on the skin in the donor section. A sample of 200 μL was extracted from the section under the skin in the Franz diffusion cell at predetermined time intervals over 5 h. The concentration of H17B was measured using a HPLC technique. However, to maintain a constant volume, a volume equal to the receptor phase was immediately applied to the Franz diffusion cell.

To evaluate the effect of iontophoresis on drug skin permeation, a current (0.5 mA/cm^2^) was created by putting Graphite Electrodes of iontophoresis on both sides of the skin (in the receptor section of the Franz diffusion cell). H17B content permeation was analysed at predominated time intervals over 30 min by HPLC as described above. The assay was performed in triplicate and the values provided are the normalized means ± SD of three independent experiments.

### 2.5 Statistical data analysis

Graph pad software was applied to calculate and analyse particle size, Zeta potential and skin permeation of nanoethosomal H17B. All triplicate experiments were repeated three times. Experiments were performed as mean ± SD. Unpaired and two‐tail Student’s t‐test was used for statistical analysis of two groups. A *p* value less than 0.05 was considered statistically significant.

## 3 RESULTS AND DISCUSSION

Uniform design is one of the most important factors in synthesising ethosomes that was proposed by Fang in 1980. In this study, in order to optimise nanoethosomes, four H17B‐ethosomes formulations were synthesised and characterised by particle size, stability, distribution and EE (Table 1) while they were visualised under SEM and TEM.

### 3.1 Stability, size and zeta potential measurements of H17B‐ethosome

One of the most important parameters to select an optimised nanocarrier is its stability over several months. Particle size is a critical factor in the determination of stability [36]. The particle size of nanocarriers is a critical characteristic in determining an efficient drug delivery. It is demonstrated that the particle size less than 300 nm might be favourably considered to permeate into deeper skin layers [37]. Therefore, theoretically, the Etho2 formulation with a particle size less than 250 nm (244 ± 4.3 nm in size and PDI = 0.368) can pass through the skin’s deep layers. The low PDI of nano‐sized ethosome is indicated in good uniformity and homogeneity of the optimised nanoethosomes [38]. Besides, Etho2 formulation showed no noticeable changes in particle size after 15 days, and it changed only 4 nm up to 30 days. Due to the high stability of Etho2 formulation, this formulation was chosen as the candidate nanoethosme in our study.

Moreover, the surface charge of ethosomes plays an important role in their stability. It is demonstrated that ethosomes with more negative surface charge are more stable. Here, the zeta potential of ethosomes was obtained at about −4.46 that could act as an electrostatic repulsion and prevent the agglomeration of ethosomes [39]. Regardless of previous studies, using 40% ethanol, which was also applied at this work, may influence the negative surface charge of ethosomes and can result in more stable ethosomes [29]. As was illustrated about how to change surface charge of ethosomes to net negative by ethanol molecules, it seems that surface ionization of ethosomes may be streamlined when terminal hydroxyl groups of ethanol molecules diffuse among the lipid bilayers; however, the expansion of hydrophilic groups to the outside medium phase may be considered as another reason. Furthermore, hydrogen bond between hydroxyl groups of ethanol with other ethanol hydroxyl groups and water molecules may facilitate ionization and negative charge of ethosomes [36, 39].

### 3.2 Characterisation by SEM and TEM

**FIGURE 2:**
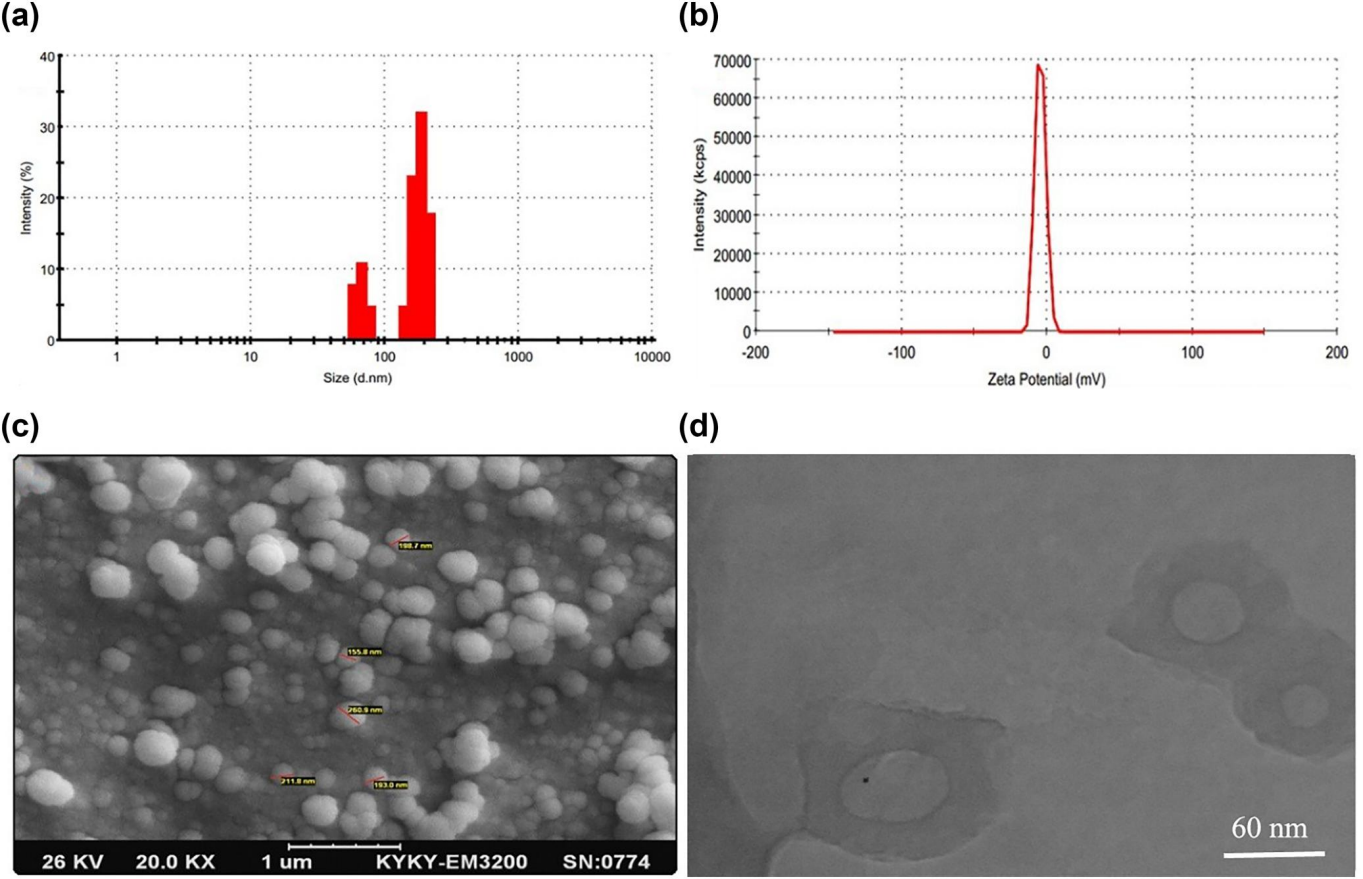
(a) The particle size and size distribution, (b) zeta potential, (c) SEM micrograph and (d) TEM of micrograph related to Etho2

Figure 2d shows a vesicle shape and smooth surface of HB17‐loaded ethosome, which is consistent with the ethosome structures reported by previous literature [39]. Additionally, the observed results of TEM images approve that the average particle size of ethosomes are around 130 nm for smaller particles. The particle size of ethosomes shown by TEM was smaller than that obtained through DLS. There may be two reasons: First, it is hard to stain and indicate the outer hydrophilic layer of ethosomes by TEM [40]; second, the hydrophilic surface of ethosomes may collapse and dehydrate during drying and staining of the TEM sample [41]. SEM images in Figure 2c demonstrate HB17‐loaded ethosome. These images confirm that the ethosomes are formed with uniform spherical structure and the average size varies from 100 to 200 nm in diameter.

### 3.3 pH analysis

The pH of formulation was measured by a pH meter at room temperature. In all synthesised HB17 loaded‐ethosomes, the amount of drug was constant (5 mg), while the ratio of other materials was changed. The results of Table 1 show that the increase of lecithin concentration leads to increased acidic power of final products. Etho2 showed pH = 7.3 ± 0.17 that was approximate to physiologic PH, which is a suitable pH for ethosomes based on previous studies. According to Table 1, the formulation Etho2, with the maximum physical stability and particle size of 244 nm was selected for skin drug delivery.

### 3.4 Entrapment efficiency (EE)% of HB17 nanoethosome

**FIGURE 3:**
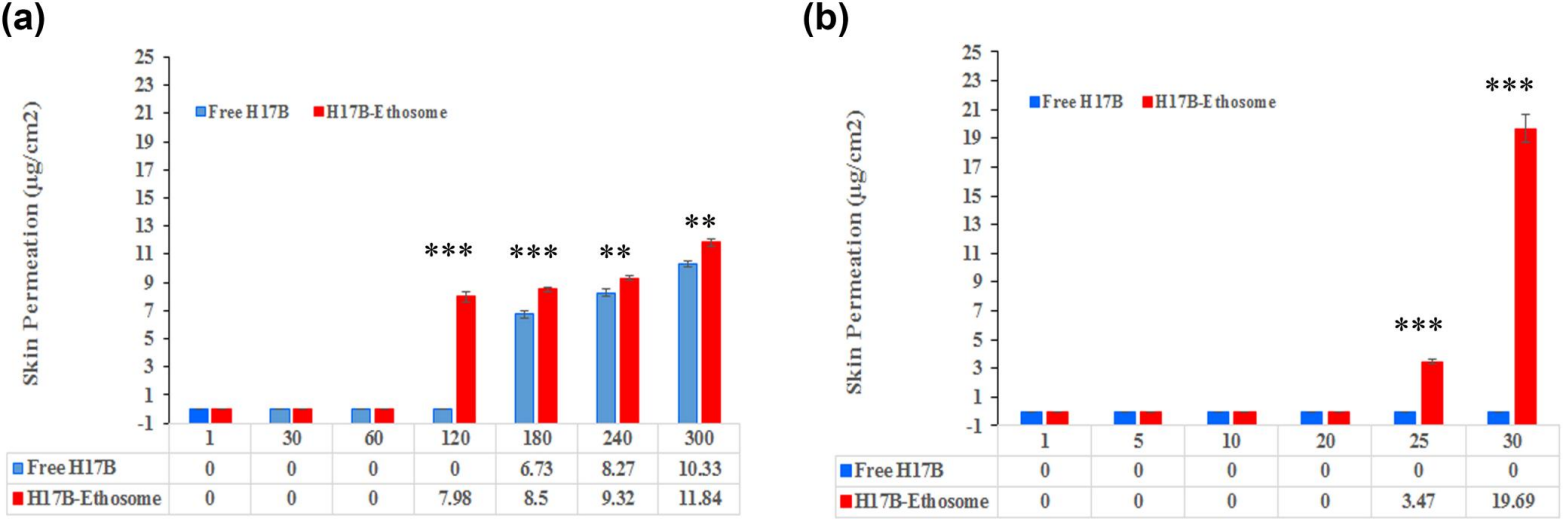
(a) Skin Permeation of HB17 from free and Etho2 nano‐ethosomal formulation at different times without iontophoresis (b) Skin Permeation of HB17 from free and Etho2 nano‐ethosomal formulations at different times with iontophoresis

**FIGURE 4:**
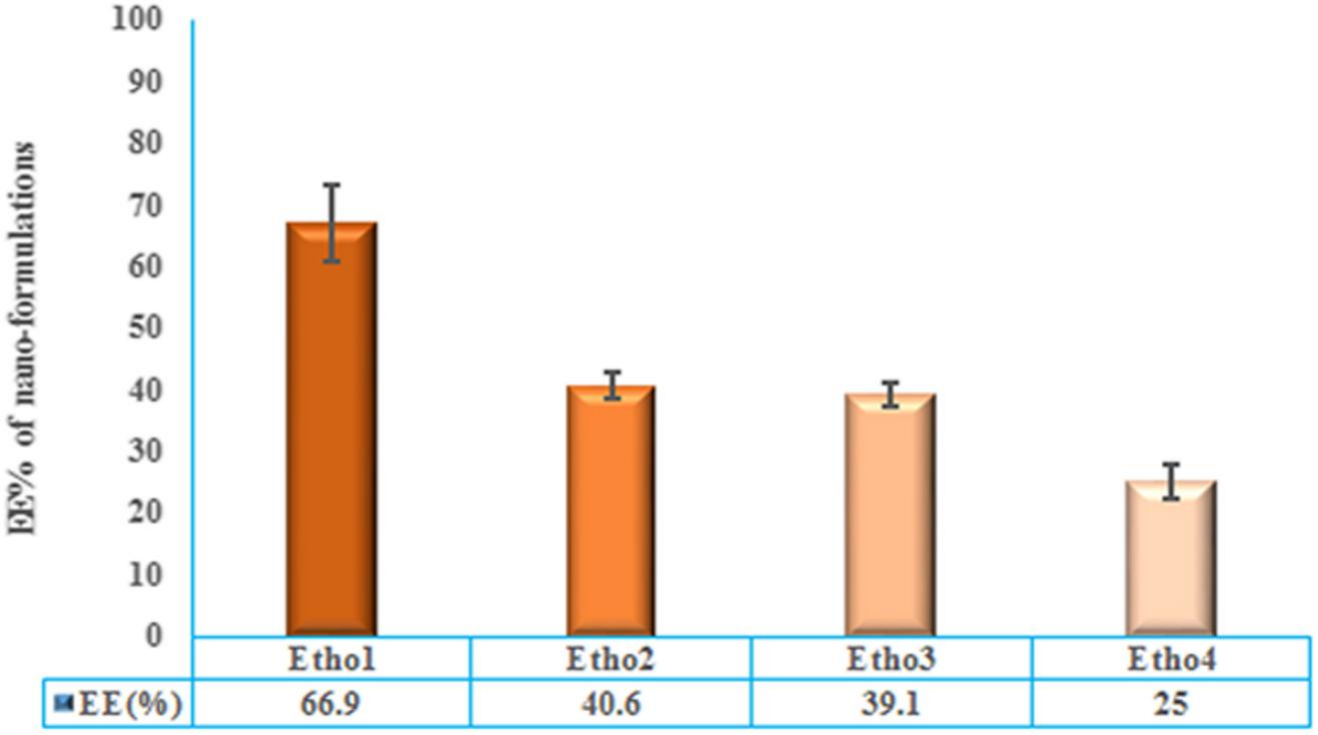
Entrapment efficiency (EE)% of different nanoformulations

EE%, as an important index in optimised formulations was measured by the HPLC technique (Figure 3a). The correlation coefficient for linearity of plot was 0.9997. The percentage of drug EE% was displayed in Figure 4. Although according to this diagram, the formulation of Etho1 has the highest EE% in comparison to another one, it did not have suitable particle size and stability. Therefore, the Etho2 formulation with EE% of 40.6 ± 2.21% was selected as the preferred candidate for transdermal drug delivery due to its high stability. Overall, an optimised formulation, including lecithin (5 mg), ethanol (10 ml) and cholesterol (0.25 mg), showed EE% of 40.6 ± 2.21. In the present work, there was a significant association between particle size and EE%; such that when size was increased, EE% was found to be elevated. Nonetheless, the sample E1 showed a slightly different finding, which might be the result of its lower stability.

### 3.5 Ex vivo skin permeation study

#### 3.5.1 Ex vivo skin permeation study without using iontophoresis

The Franz diffusion cell was used to ex vivo study the speed and permeation depth of free and loaded HB17 into the complete‐thickness rat skin with an effective penetration region of 3 cm^2^. Here, the permeation speed of HB17‐loaded ethosomes and free HB17 was measured by the HPLC system; the results showed a significant increase in the penetration speed of HB17‐loaded ethosomes into the full‐thickness rat skin as compared to free HB17 (Figure 3a). As Figure 3a shows, HB17‐loaded ethosomes and free HB17 started to permeate into the full‐thickness rat skin at 120 and 180 min, respectively. Overall, there are no significant differences between the percentage of the HB17‐loaded ethosomes crossing through the full‐thickness rat skin (11.84 ± 2.63%) than free HB17 (10.33 ± 2.93%) during 300 min (*p* value = 0.5429). Results indicated the effectiveness of ethosomes in skin drug delivery at early stages. It is suggested that the penetration of ethosomes into the skin may be related to impressive interaction of ethosomes with the skin. Furthermore, enhancing depth and speed penetration of ethosomes into the skin may depend on colloidal features of these compounds [39]. Flexibility and deformability of ethosomes increase by the presence of ethanol and it may facilitate crossing HB17‐loaded ethosomes into the skin channels through a self‐adapting process [42]. Furthermore, fast partitioning of ethosomes into the stratum corneum had been proposed as a reason for facilitating and improving drug transport into the skin by ethosomes. Another factor that may be assumed as a reason, is the solubility of HB17 in the vesicles derived from ethanol. Moreover, changing the solubility properties of the tissue by the penetration of ethanol could improve HB17 partitioning into the membrane [43]. Additionally, two substantial parts of ethosomes, which may be involved in improving penetration by changing the lipid structures are ethanol and lecithin. The former is able to disturb the lipid bilayer's organization in the stratum corneum by extracting some of the lipid fraction of stratum corneum to improve HB17 flux through the skin [44]. The latter could mix with lipids of skin by exchanging lipids and disorganising the lamellar alignment of the lipids in stratum corneum [45]. Overall, the above mentioned two mechanisms can act synergistically and/or separately as the main mechanism for quicker skin penetrability of HB17 than the free drug.

#### 3.5.2 Ex vivo skin permeation study using iontophoresis

Over the decades, iontophoresis has been investigated as a suitable method to improve transdermal delivery utilising perpetually weak voltage current [46]. This improvement can be induced by two phenomena: 1. Permeability of skin can be increased by iontophoresis, 2. Transporting through stratum corneum using an electrical moving force made by iontophoresis. There are two common ways to transfer molecules through stratum corneum such that charged structures are moved using electrophoresis, whilst other compounds including low charged and/or uncharged ones move along with electroosmotic flux in water [47]. Interestingly, as presented in Figure 3b, the quantity of delivered HB17 indicated a substantial increase from zero to 19.69 ± 4.57% using iontophoresis during 0–13 min. Similarly, the transdermal flow as well as the quantity of the drug increased as compared to the free drug, which did not show any difference in the amount of delivered drug (zero in both 1 and 30 min). These results were in line with other researchers who have shown that the iontophoresis system can be only applied as an especial instrument for transdermal administration of hydrophobic, charged and small structures because iontophoresis is not able to alter the skin instinctive obstacles [14, 48]. Despite substantial increase in drug delivery and transdermal flux, as valuable advantages of iontophoresis, there are some big worries about iontophoresis; High voltage current may damage the skin, so efficient electrochemical stability of ethosomes needed to be evaluated under the iontophoresis system, where pores dilated in the lipid bilayers may be created by the electric current, which could lead to the drug's leakage due to irreversible breakdown of ethosomes [49]. However, the amount of current electric, which could create pore widening and irreversible breakdown of vesicles was reported to be more than 0.5 mA/cm^2^; but in this work, we used the 0.5 mA/cm^2^ current, which may not be sufficient to induce rupture in ethosomes.

## 4 CONCLUSION

Prescription of anti‐inflammatory drugs may be considered as a promising strategy in chronic wound healing where the inflammatory disturbance has delayed the healing process. It seems that corticosteroids such as HB17 would be promising in the form of a nanoformulation to quicker adsorb and decline inflammation. The potential advantages of the formulation include; (i) quicker delivery, (ii) simple preparation and lower cost and (iii) propensity for improved patient convenience and compliance with resultant better therapeutic outcomes [50]. In summary, the ethosomal HB17 consisting lecithin (2.5–20 mg), ethanol (10 ml), cholesterol (0.125–1 mg) and water (25 ml) was successfully synthesised by the hot method. Based on the results, the E2 formulation, with the particle size of 244 nm showed no considerable change in size up to 30 days. It also had the entrapment efficiency of 40.63% and hollowed spherical morphology; thereby, this formulation of ethosome was chosen as the candidate formulation. However, the results of ex‐vivo test demonstrated a gentle increase in the speed of H17B penetration (7.98 μg/cm^2^ in 120 min) when the formulation was used for H17B treatment in the rat skin as compared to free H17B (zero at the same time). The synergistic mechanism between ethanol, surfactant, vesicles and skin lipids are responsible for a quicker skin penetration of ethosomes [51]. On the other hand, a combination of ethosomes and iontophoresis showed a sharp increase in the speed and amount of H17B permeation (19.69 μg/cm^2^ in 30 min) into the rat skin. It might be concluded that ethosomal H17B may be considered as a promising drug nanocarrier in patients with chronic wounds; however, iontophoresis significantly enhances its drug delivery efficacy.

## CONFLICT OF INTEREST

Authors declare no conflict of interest.

## Data Availability

Data will be available upon request.
